# Clinical audit in the pediatric primary care office and overweight prevention in toddlers

**DOI:** 10.1186/s12887-020-02076-y

**Published:** 2020-04-14

**Authors:** Raffaele Limauro, Patrizia Gallo, Luigi Cioffi, Angelo Antignani, Valentina Cioffi, Patrizia Calella, Giuliana Valerio

**Affiliations:** 1 FIMP (Italian Federation Pediatricians), Naples Section, Pediatric Primary Care Local Health Authority NA 3 Sud, P.co Carelli 23, 80123 Naples, Italy; 2 School of Specialization in Human Nutrition, AOU Federico II, Naples, Italy; 3 Master of Science in Biology, Department of Biology, University of Naples Federico II, Naples, Italy; 4 Department of Movement Sciences and Wellbeing, University of Naples Parthenope, Naples, Italy

**Keywords:** Children, Clinical audit, Feeding, Overweight, weaning

## Abstract

**Background:**

Clinical audit is a process by which physicians or other health care professionals perform a regular and systematic review of their clinical practice and amend it, when necessary. An internal audit allows to review the activities carried out by professionals, in order to assess the appropriateness, effectiveness, efficiency and safety of the services provided. Aim of this study was to apply the process of clinical audit to the obesity/overweight care in toddlers. After the correction of the nutritional errors that were considered potentially responsible for the excess weight gain, the effect of the changes of dietary advice on the frequency of overweight/obesity was assessed in a cohort of children aged 24–36 months.

**Methods:**

Three Italian primary care pediatricians set up the audit strategy by recognizing the high prevalence of overweight and obesity in the entire cohort of toddlers born in 2005, 2006 and 2007 (Pre-Audit group, age 24–36 months old) under their care. By reviewing their clinical practice, they changed the protocol of weaning and feeding up to 36 months, mainly reducing protein and sugar excess. The change involved the cohorts of toddlers born in the years 2010, 2011 and 2012 (Post-Audit group).

**Results:**

Change in the approach of pediatricians to children’s diet yielded a reduction of the frequency of overweight/obesity in children between 24 and 36 months of life from 26.3% in the Pre-Audit group to 13.9% in the Post-Audit group (*p* < 0.0001).

**Conclusion:**

Clinical audit revealed high rates of obesity/overweight among toddlers. The practice developed a new strategy for nutritional counseling, which was effective in reducing the frequency of overweight/obesity in young children.

## Background

Clinical audit is a process by which physicians, nurses or other health care professionals perform a regular and systematic review of their clinical practice and amend it, when necessary [1]. This revision process, based on established criteria, has the main purpose to evaluate the efficacy, effectiveness, and safety of medical care. The final report provides indications aimed to improve medical care [2]. The availability of electronic medical records represents an important source of data for audits.

An audit process that investigates the possible nutritional errors responsible of overweight and obesity in young children may be an opportunity for the appraisal of patient care, implementing specific educational measures considered for improvement. Indeed, childhood obesity represents one of the most important health issue worldwide. Specifically, the prevalence of overweight and obesity in the South of Italy (Campania region) is the highest in the country, as assessed by the Nutritional Surveillance system “Okkio alla Salute”. According to the last estimate in the 2016 [3] the prevalence of overweight and obesity in children living in the Campania region amounted to 44.1% (26.2% overweight, 17.9% obesity). In early life, nutritional factors play a greater role on weight gain than other environmental factors, such as physical activity, and are associated to adiposity increase from the prenatal age through the first 36 months of life [4]. Therefore, it is indispensable that pediatricians carefully revise their recommendations about complementary feeding and diet in the first 36 months of life. In particular, beyond sugar overconsumption [5], the excessive protein intake, especially of lacto-proteins, has been identified as a risk factor for the premature development of obesity [6–9] leading to early adiposity rebound [10, 11]. On the other side, a correct protein intake may be protective [12].

Since translating nutritional guidelines into clinical practice often needs a long processing time to ensure the optimal care of children, audit may be used to improve the quality of care leading to small but potentially important enhancements in the medical care [13]. As all the primary care pediatricians in Italy are provided with a medical software to store the demographic and clinical data of their child population, the revision of their clinical practice is facilitated.

Our study was conducted in the Pediatric primary care office setting in the Campania region and was aimed at assessing the impact of the clinical audit on the evaluation of the possible nutritional errors underpinning the excess weight and measure the effect of the changes of dietary advice on the frequency of overweight/obesity in children aged 24–36 months.

## Methods

This is as a pre-post pilot study without a control group. The audit was set up in 2009 by three primary care pediatricians working in Naples (Campania region, South Italy). They assessed the frequency of overweight and obesity in the entire population of children aged 24–36 months old (corresponding to the birth years 2005, 2006 and 2007) under their care, using the anthropometric measures available in their electronic databases. They found that 26.3% toddlers (123 out of 467) were overweight or obese (Pre-Audit Group) (Table 1).

**Table 1:** Demographic and anthropometric data of children included in the Pre-Audit and in the Post-Audit groups at the age of 24–36 months, according to the birth year

	Pre-audit group Birth year			Post-audit group Birth year		
	2005	2006	2007	2010	2011	2012
Number	135	136	196	155	146	165
Boys/girls	71/64	66/70	104/92	78/77	70/76	87/78
Age, months	30.7 ± 2.0	30.7 ± 2.9	31.7 ± 3.4	30.7 ± 3.2	29.2 ± 3.1	30.2 ± 2.6
Weight, kg	14.3 ± 2.4	14.3 ± 2.3	14.5 ± 1.8	14.1 ± 1.9	13.6 ± 1.8	13.8 ± 1.6
Length/height, cm	91.1 ± 9.2	92.3 ± 4.1	92.8 ± 4.1	91.9 ± 3.8	90.8 ± 3.1	91.3 ± 3.7
BMI, kg/m^2^	16.6 ± 2.2	16.8 ± 1.5	16.7 ± 1.7	16.6 ± 3.3	16.4 ± 1.3	16.3 ± 1.5
Normal weight, n (%)	96 (71.1)	108 (79.3)	140 (71.4)	130 (83.9)	129 (88.3)	142 (85.7)
Overweight, n (%)	20 (14.8)	18 (13.2)	33 (16.8)	11 (7.1)	11 (7.5)	20 (10.2)
Obesity, n (%)	19 (14.1)	10 (7.4)	23 (11.8)	14 (9.0)	6 (4.1)	3 (4.0)
Overweight/Obesity, n (%)	39 (28.9)	28 (20.6)	56 (28.6)	25 (16.1)	17 (11.6)	23 (14.2)

**Fig. 1:**
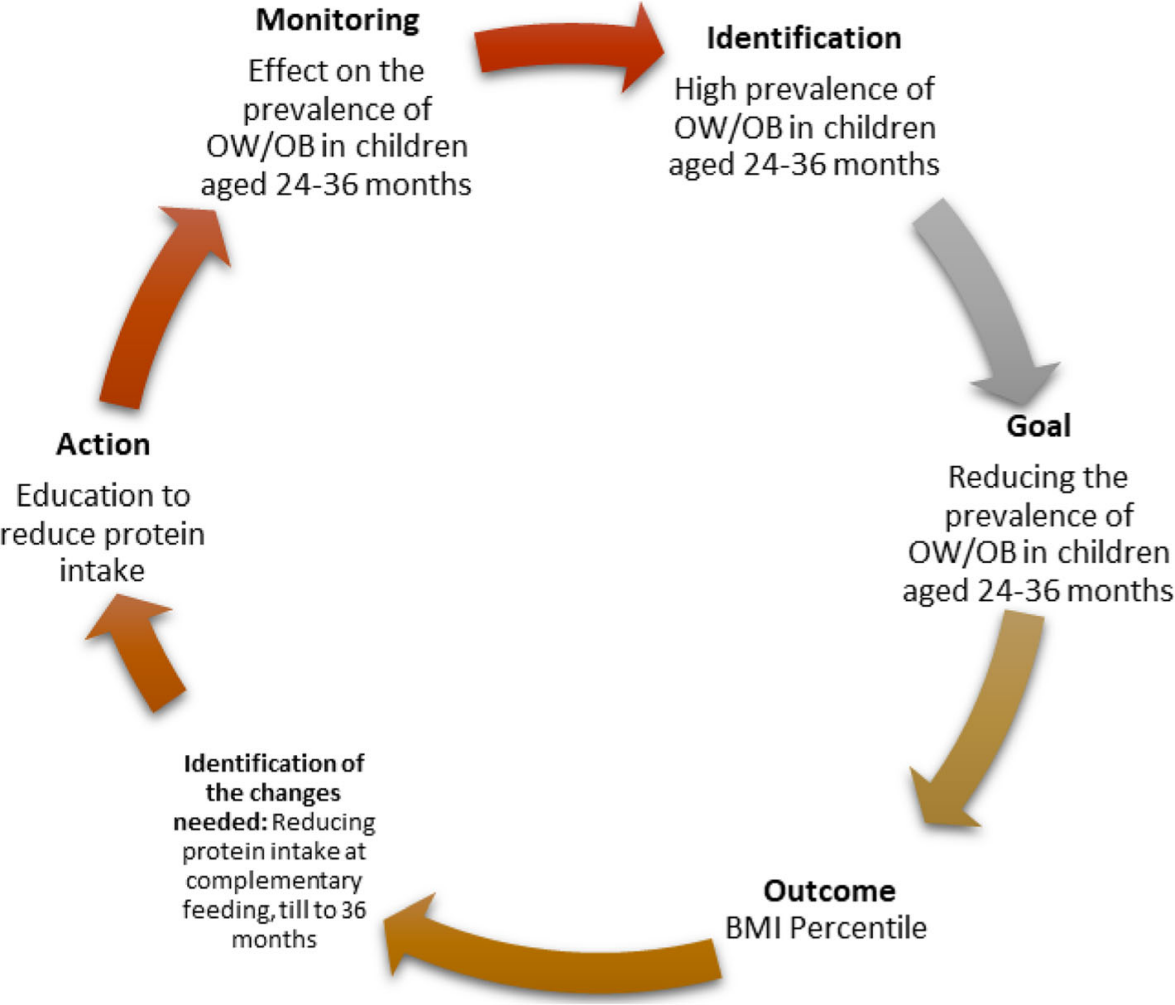
The schematic representation of the clinical audit cycle

The clinical audit was conducted through four main stages, which are synthetized in Fig. 1. The first stage corresponds to the identification of the problem. Possible nutritional causes of excessive caloric intake in toddlers were hypothesized on the basis of the literature. The pediatric approach regarding complementary diet and diet recommendations up to 36 months of life was discussed and the possible errors were identified (Table 2).

**Table 2:** Feeding recommendations from 6 to 36 months in the Pre- and Post-Audit periods

Pre-Audit period	Post-Audit period
Raw meat or fish 30 g per meal from weaning to 12 months	Raw meat or fish 15 g per meal from weaning to 12 months
No information that legumes and parmesan cheese^a^ are protein source and excellent substitutes of meat and fish	Education about protein food sources. Advise to alternate vegetable and animal food proteins (legumes 3–4 times/week; fish 3–4 times/week).
Breast milk until 24 months or cow milk after the age of 12 months (protein content roughly 3.3 g/100 ml)	Breast milk until 24 months or formula until 36 months of age (protein content roughly 1.4–1.6 g/100 ml)
No information that the second course (commercial or fresh baby food) needs to be weighed after the age of 12 months	Commercial baby food 80 g or 30 g raw meat or fish per meal after the age of 12 months
No specific advice to avoid sugar excess	Specific advice to avoid food high in sugar and sugar-sweetened beverages

^a^ the use of this kind of cheese, which is far higher in protein-density than other types of cheese, is a consolidate habit among the mothers of very young children

In the second stage pediatricians prefixed the goal of reducing the frequency of overweight/obesity in the cohorts of 24–36 months children (birth years: 2010–2011-2012) (Post-Audit Group) (*Goal*). To this purpose, they drafted a shared protocol of weaning and feeding up to use until the age of 36 months, which was more specifically focused on education about protein content of toddlers’food, reduction of the animal protein sources and discouragement of sugar and sugar-sweetened beverages [14] (Table 2). In the third step (*Action*) the updated recommendations were addressed by means of written instructions to the parents of children of the Post-Audit Group, starting at the time of weaning (around the 6th month of life), and progressively continuing up to age of 36 months. Parents’dietary behaviour was assessed at every well-child visit. In the last step, the effect of change on the body mass index (BMI) was monitored through the assessment of the prevalence of overweight and obesity in the Post-Audit group of children at 24–36 months of life (*Monitoring*). The study was approved by the Ethical Committee “Campania Sud” of the ASL Napoli 3 Sud and was performed in accordance with the 1975 Declaration of Helsinki revised in 1983. To ensure data protection and confidentiality, data extracted from the medical records were anonimyzed before being uploaded in a database for analyses.

### Anthropometric measures

Body weight and height were measured in each office by the same pediatrician, who was specifically trained in anthropometry, according to standard procedures [15]. The same procedures were followed both in the Pre-Audit and in the Post-Audit toddlers. Body weight was determined to the nearest 0.1 kg on standard physician’s beam scales (SECA 756, UK), with the subject wearing only underwear and no shoes. Height was measured to the nearest 0.1 cm using a stadiometer (SECA 213, UK). Measures of height were taken three times and the mean value was considered for data analysis. BMI (kg/m^2^) was calculated as body weight divided by the square of height. Categories of normal weight (NW), overweight (OW) and obesity (OB) were defined according to the BMI thresholds proposed by the Centers for Disease Control and Prevention [16].

### Statistical analyses

Statistical analyses were carried out using the Statistical Package of Social Sciences (SPSS, Chicago, IL, USA) for Windows software program release 15.0. A *p* value < 0.05 was considered statistically significant. Results are reported as mean and standard deviations or as absolute and relative frequencies. The difference in the prevalence of overweight and obese children between the Pre-Audit Group and Post-Audit Group was tested by the Pearson chi-square test. Subgroup analyses were performed separately in the three pediatrician’s offices and in the whole sample. The relative risk (RR) adjusted for confounders (gender and age) and the number needed to treat (NNT) were calculated in the whole sample by using stratified analysis of 2 × 2 Tables, using the Epi Info for Windows StatCalc software.

## Results

Demographic and anthropometric data of the Pre-Audit and Post-Audit groups assessed at the age of 24–36 months stratified according to each pediatrician’s office are shown in Table 3. No difference in gender distribution or prevalence of overweight/obesity was found among children followed by the three pediatricians both in the Pre-Audit and Post-Audit cohorts. A significant reduction in the overall prevalence of overweight and obese children in the Post-Audit group compared to the Pre-Audit-Group was found for each practitioner (Table 3).

**Table 3:** Demographic and anthropometric data of children included in the Pre-Audit and in the Post-Audit groups at the age of 24–36 months in the three pediatricians’offices

	Pediatrician A		Pediatrician B		Pediatrician C	
	Pre-audit	Post-audit	Pre-audit	Post-audit	Pre-audit	Post-audit
Number	155	155	158	165	154	146
Boys/girls	78/77	78/77	83/75	81/84	80/74	68/78
Age (months)	30.8 ± 3.34	29.3 ± 2.8	30.4 ± 2.2	29.5 ± 3.3	28.6 ± 3.8	29.8 ± 4.2
Weight (kg)	14.1 ± 1.9	13.9 ± 1.9	14.2 ± 2.3	13.7 ± 1.9	13.9 ± 1.9	13.7 ± 1.5
Length/height (cm)	91.5 ± 7	91.3 ± 3.8	91.9 ± 5.2	90.9 ± 4.1	91.4 ± 4.2	91.8 ± 3.6
BMI (kg/m^2^)	16.8 ± 1.6	16.6 ± 1.5	16.7 ± 1.7	16.4 ± 2.1	16.6 ± 1.5	17.4 ± 1.3
Normal Weight n (%)	111 (71.6)	135 (87.1)	119 (75.3)	141 (85.4)	114 (74)	125 (85.6)
Overweight n (%)	28 (18.0)	13 (8.4)	19 (12.0)	15 (9.1)	24 (15.6)	14 (9.6)
Obesity n (%)	16 (10.4)	7 (4.5)	20 (12.7)	9 (5.0)	16 (10.4)	7 (4.8)
Overweight/Obesity n (%)	44 (29.2)	20 (12.9)	39 (24.7)	24 (14.6)	40 (26.0)	21 (14.4)
Normal weight vs overweight	*P* < 0.01		*P* = 0.2		P 0.05	
Normal weight vs obesity	*P* < 0.02		P < 0.01		*P* = 0.05	
Normal weight vs overweight/obesity	*P* < 0.001		P < 0.02		*P* < 0.01	

**Fig. 2:**
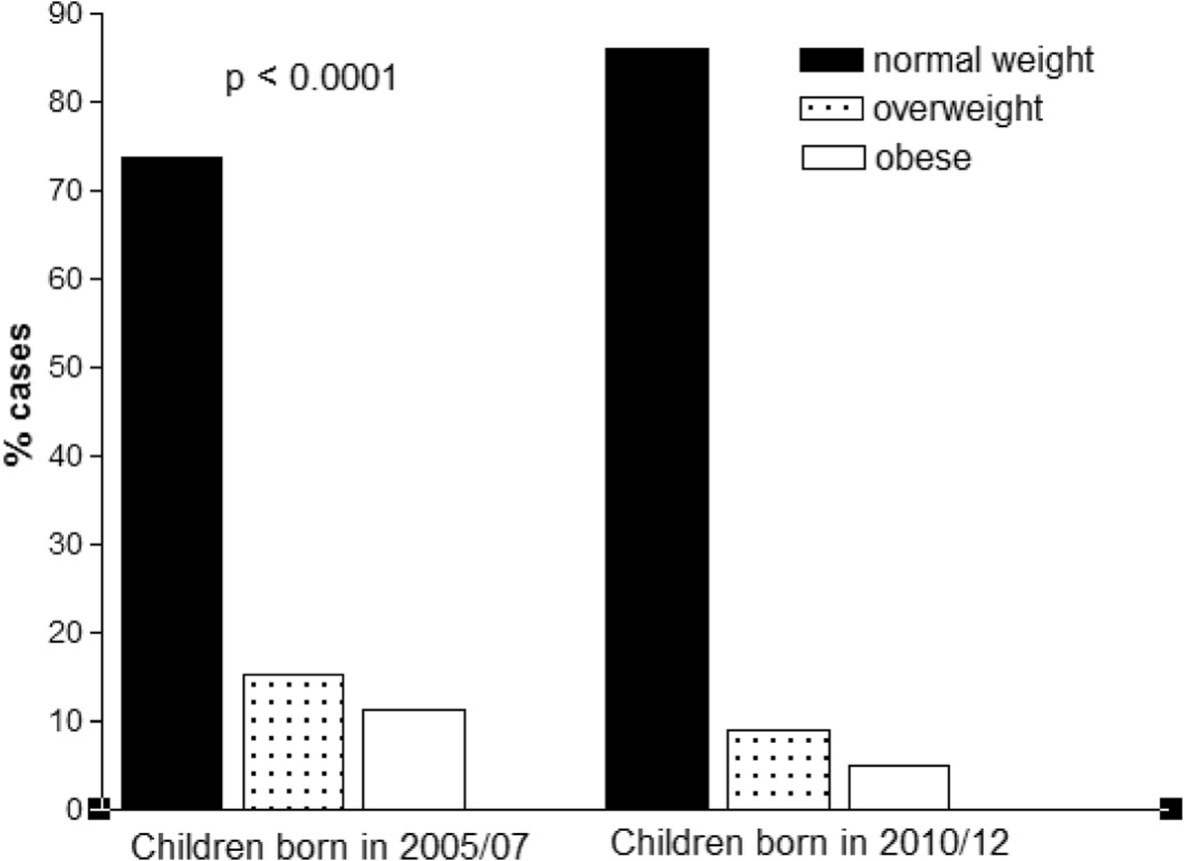
Prevalence of normal-weight, overweight and obesity in the entire cohort of toddlers in the Pre-Audit and Post-Audit groups

The overall prevalence of overweight and obesity in the entire cohort of the Pre-Audit and Post-Audit cohorts is shown in Fig. 2. Specifically, reductions of 60% in the rate of overweight and 44% in the rate of obesity were found in the Post-Audit Group (*p* < 0.0001). After adjusting for confounders (age and gender), the RRs for overweight or obesity were 7.63 (Confidence interval 3.09–12.16) and 7.71 (Confidence interval 3.74–11.67), respectively in the Pre-Audit cohorts with respect to the Post-Audit cohorts. The NNT was 14 for overweight and 13 for obesity.

## Discussion

Our results have shown that the clinical audit is an effective tool in identifying inaccuracies in medical procedures and helpful in changing attitude when required, leading to positive outcomes on the quality of healthcare. Precisely, simple but specific changes in the dietary advice were associated to reduction of the frequency of overweight and obesity in children between 24 and 36 months of age, living in an Italian region with high prevalence of overweight and obesity.

A clinical report from the American Academy of Pediatrics supports the role of Pediatricians in the primary prevention of obesity [17]. It has been demonstrated that the first 1000 days of life, the period from conception to age of two, represent the best time for obesity prevention [18]. Several observational or randomized controlled trials have demonstrated that higher protein content in in the first two years of age is responsible of higher postnatal growth velocity and an early adiposity rebound, which is predictive of greater fatness in the following years [19, 20]. Pediatricians should be properly informed of the complex and interrelated factors that lead to excessive weight gain in this specific period of life and improve their ability to provide recommendations that are relevant and evidence based to the families.

Clinical audit led pediatricians to revise their procedures about nutritional counselling and make relevant changes that involved dietary intake, evaluation of food literacy and misconceptions regarding infant feeding among the parents. Specifically, following the changes in the pediatricians’ procedures about the choice and amount of high protein food, it was estimated a reduction of protein intake from more than 3.5 g/kg to about 2 g/kg from 13 to 36 months [6]. In Southern Italy, only 29.1% children are breastfed at the age of 12 months, while the majority is fed with cow milk, 250 mL twice a day [21]. Therefore, the protein intake from milk is generally equal to 16.5 g, which adds to other proteins provided from meats, fish and eggs. Although the amount of 2 g/kg of proteins is still far from the recommended reference levels of nutrient and energy intake for the Italian population (LARN) [22], we preferred to avoid drastic changes in the family habits in order to obtain greater compliance. In anycase, protein intake was far less than the value of 3.5 g/kg, which is deemed to be a risk factor for lipogenesis and fat mass gain in early life [6].

The choice to consider a straightforward, easy to understand approach has proved to be effective, despite the NNT was still high; on the other hand, providing dietary advice based upon the best available evidence is among the duties and responsibilities of primary care pediatricians, hence it doesn’t represent a further burden in their practice [23]. This study had some limits, such as the low number of pediatricians involved, the choice of considering only a few determinants (protein and sugar excess) despite the multifaceted aspects of the pathology and the lack of a parallel control group. Considering these limits, a new study has been designed on a larger sample of children, in whom the effects of the change in nutritional procedures will be compared to an appropriate control group, and the influence of the other determinants of excess weight gain in early childhood, such as birth weight, parental obesity and the family socioeconomic level will be considered. The strength of the study was the implementation of evidence based procedures in the primary care setting and the immediate transferability of this procedure among pediatricians. The availability of electronic clinical data of children, followed in the pediatric primary care office setting, has enabled the clinical audit.

## Conclusions

The first three years of life are a critical time period where parents are educated to establish healthy feeding patterns that may help to restrain the obesity trend. This pilot study, notwithstanding its simplicity, clearly showed that clinical audit has provided the opportunity of self-evaluation of the pediatricians’ healthcare procedures about feeding in the first years of age and assessment of the effect of change on the prevalence of overweight and obesity. Audit may have an educational value as it creates opportunities for professionals to think about their practice and to learn from the experience of others. It represents also a useful starting point to plan studies involving a larger number of practitioners and patients, in order to verify the effectiveness of interventions.

## Data Availability

Datasets used and/or analyzed during the current study are available from the corresponding author on reasonable request.

## Abbreviations

BMI: Body mass index
NNT: Number needed to treat
RR: Relative risk

## References

[1] Baker R (1995). Clinical audit in primary health care: towards quality assurance. BMJ..

[2] Hoelscher DM, Kirk S, Ritchie L, Cunningham-Sabo L (2013). Position of the academy of Nutrition and dietetics: interventions for the prevention and treatment of pediatric overweight and obesity. J Acad Nutr Diet.

[3] Basso D, Avolio M, Sabetta T, Longhi S, Marino M, De AG (2013). The italian situation of overweight and obesity in childhood: a problem with epidemic proportions in the world. Aten Primaria.

[4] Lanigan J (2018). Prevention of overweight and obesity in early life. Proc Nutr Soc.

[5] Pan L, Li R, Park S, Galuska DA, Sherry B, Freedman DS (2016). A longitudinal analysis of sugar-sweetened beverage intake in infancy and obesity at 6 years. World Rev Nutr Diet.

[6] Hörnell A, Lagström H, Lande B, Thorsdottir I (2013). Protein intake from 0 to 18 years of age and its relation to health: a systematic literature review for the 5th Nordic Nutrition recommendations. Food Nutr Res.

[7] Appleton J, Russell CG, Laws R, Fowler C, Campbell K, Denney-Wilson E (2018). Infant formula feeding practices associated with rapid weight gain: a systematic review. Matern Child Nutr.

[8] Pimpin L, Jebb S, Johnson L, Wardle J, Ambrosini GL (2016). Dietary protein intake is associated with body mass index and weight up to 5 y of age in a prospective cohort of twins. Am J Clin Nutr.

[9] Luque V, Closa-Monasterolo R, Escribano J, Ferré N (2015). Early programming by protein intake: the effect of protein on adiposity development and the growth and functionality of vital organs. Nutr Metab Insights.

[10] Rolland-Cachera MF, Akrout M, Péneau S (2016). Nutrient intakes in early life and risk of obesity. Int J Environ Res Public Health.

[11] Eriksson JG, Forsén T, Tuomilehto J, Osmond C, Barker DJP (2003). Early adiposity rebound in childhood and risk of type 2 diabetes in adult life. Diabetologia..

[12] Weber M, Grote V, Closa-Monasterolo R, Escribano J, Langhendries JP, Dain E (2014). Lower protein content in infant formula reduces BMI and obesity risk at school age: follow-up of a randomized trial. Am J Clin Nutr.

[13] Ivers N, Jamtvedt G, Flottorp S, Young JM, Odgaard-Jensen J, French SD (2012). Audit and feedback: effects on professional practice and healthcare outcomes. Cochrane Database Syst Rev.

[14] Oddy WH (2012). Infant feeding and obesity risk in the child. Breastfeed Rev.

[15] Gallo P, Cioffi L, Limauro R, Farris E, Bianco V, Sassi R (2016). SGA Children in Pediatric Primary Care: What Is the Best Choice, Large or Small? A 10-Year Prospective Longitudinal Study. Glob Pediatr Heal.

[16] Kuczmarski RJ, Ogden CL, Guo SS, Grummer-Strawn LM, Flegal KM, Mei Z (2002). 2000 CDC growth charts for the United States: methods and development. Nationale Center Health Stat.

[17] Daniels SR, Hassink SG, Committee on nutrition (2015). The role of the pediatrician in primary prevention of obesity. Pediatrics..

[18] Blake-Lamb TL, Locks LM, Perkins ME, Woo Baidal JA, Cheng ER, Taveras EM (2016). Interventions for childhood obesity in the first 1,000 days a systematic review. Am J Prev Med.

[19] Voortman T, Braun KVE, Kiefte-De Jong JC, Jaddoe VWV, Franco OH, Van Den Hooven EH (2016). Protein intake in early childhood and body composition at the age of 6 years: the generation R study. Int J Obes.

[20] Hörnell A, Lagström H, Lande B, Thorsdottir I (2013). Protein intake from 0 to 18 years of age and its relation to health: a systematic literature review for the 5th Nordic Nutrition recommendations. Food Nutr Res.

[21] ISTAT Gravidanza, parto e allattamento al seno. Anno 2013. https://www.istat.it/it/files//2014/12/gravidanza.pdf.

[22] SINU. Livelli di Assunzione di Riferimento di Nutrienti ed energia per la popolazione italiana. Sintesi prefinale. 2012. http://www.salute.gov.it/imgs/C_17_dossier_26_listaFile_itemName_0_file.pdf.

[23] Valerio G, Maffeis C, Saggese G, Ambruzzi MA, Balsamo A, Bellone S (2018). Diagnosis, treatment and prevention of pediatric obesity: consensus position statement of the Italian Society for Pediatric Endocrinology and Diabetology and the Italian Society of Pediatrics. Ital J Pediatr.

